# Larger-volume capillary blood sampling is a valid alternative to assess progesterone and 17ß-estradiol for cycle phase identification in tactical female athletes

**DOI:** 10.3389/fphys.2025.1743330

**Published:** 2026-01-13

**Authors:** Jennifer Schlie, Eva Fellinger, Sina Albrecht, Jessica Güldenstern, Annette Schmidt

**Affiliations:** 1 Institute for Sports Science, Chair of Sports Biology, University of the Bundeswehr Munich, Neubiberg, Germany; 2 dtec.bw, NextGenerationEU Project Smart Health Lab, University of the Bundeswehr Munich, Germany, Chair of Sport Biology, GER, Neubiberg, Germany; 3 Kompetenzzentrum für Funktionsfitness, University of the Bundeswehr Munich, Neubiberg, Germany

**Keywords:** cycle phase verification, ELISA, estrogen, female physiology, female sex hormones, follicular phase, luteal phase

## Abstract

**Introduction:**

Serum-based hormone analysis is considered essential for determining menstrual cycle phases in sport and exercise science. However, its reliance on venous blood sampling limits applicability in field-based or operational contexts. This study evaluated the validity of larger-volume capillary samples obtained from the earlobe for the quantification of progesterone (P4) and 17ß-estradiol (E2) in comparison to venous blood sampling.

**Materials and method:**

Twelve eumenorrheic female soldiers (mean age: 24.4 ± 2.9 years; BMI: 24.4 ± 2.2 kg/m^2^) participated in a longitudinal protocol involving paired capillary and venous blood sampling twice weekly across one complete individual menstrual cycle. Blood was drawn from the earlobe (capillary, 100–250 µL) and antecubital vein (venous, 4 mL) and analyzed via ELISA for P4 and E2 concentrations.

**Results:**

All participants completed six or more sampling timepoints and had ovulatory cycles, with a mean cycle length of 28.3 ± 3.6 days and ovulation occurring on day 16.6 ± 4.7. On average, P4 concentrations were 1.6 ng/mL higher in venous compared to capillary samples, while E2 values were 0.34 pg/mL lower. The concordance correlation coefficients were 0.911 for P4 and 0.919 for E2, indicating good to very good agreement between the sampling methods. Repeated measures Bland-Altman analysis with mixed effects revealed minimal mean bias for both hormones, with acceptable limits of agreement. Repeated measures correlation coefficients were 0.915 and 0.982 for E2 and P4, respectively.

**Discussion and conclusion:**

The results demonstrate that earlobe-derived capillary sampling is a valid and practical alternative to venous sampling for hormonal assessment across the menstrual cycle. The method yielded robust results for both P4 and E2, with sufficient accuracy to support cycle phase classification and the detection of anovulatory or luteal-phase deficient cycles. The logistical advantages include minimal invasiveness, no need for medical personnel, and the ability to analyze the frozen samples at a later date. This makes capillary sampling particularly well suited for use with athletes and tactical populations. Future studies should explore its application in elite athletes and incorporate participant-reported burden to optimize feasibility in high-frequency sampling protocols.

## 1 Introduction

The influence of different menstrual cycle phases on physical performance is a topic of constant scientific investigation that remains insufficiently understood (Schlie et al., 2025). While it has recently received increasing attention in sport and exercise science, its relevance extends beyond athletic settings to physically demanding occupations such as military service, firefighting, and law enforcement, where physical readiness is essential. Although more and more females enter these formerly male-dominated professions, there is still few scientific research on women in the military, especially with regard to the menstrual cycle (Treg et al., 2023). Female soldiers must ensure physical readiness regardless of their current cycle phase, similar to elite athletes who have to deal with set competition schedules. In addition, female soldiers suffer from musculoskeletal injuries at a significantly higher rate than their male counterparts (O’Leary et al., 2023; Knapik et al., 2012). Therefore, cycle-related hormone fluctuations could have important practical implications in the future of tactical populations. They may represent a modifiable risk factor in injury prevention and performance enhancement, underscoring the need for well-informed research in this field (Treg et al., 2023).

A crucial first step in understanding and investigating potential effects of the menstrual cycle on performance is the accurate identification of distinct hormonal phases throughout the menstrual cycle.

**FIGURE 1:**
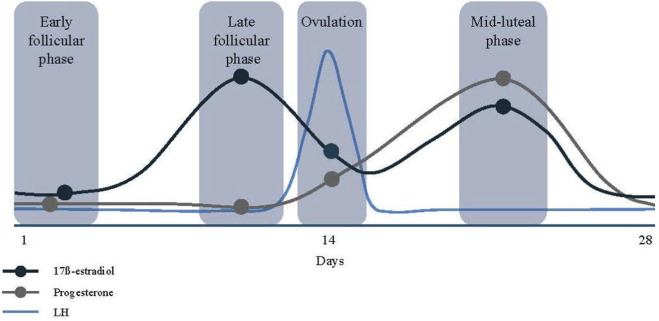
Graphical representation of the hormonal changes over an idealized 28-day menstrual cycle (adapted from Schlie et al. (2025)). 17ß-estradiol (E2) shown in black, progesterone (P4) in grey, luteinizing hormone (LH) in blue. Four distinct menstrual cycle phases are shown: the early follicular- and late follicular phase, ovulation, and the mid-luteal phase. The respective concentrations of E2 and P4 for the different phases are highlighted with circles.

In sport and exercise science, two major methodological questions are widely discussed: 1) Which phases of the cycle should be differentiated? While many studies to date have only distinguished between the follicular and luteal phases, recent consensus recommendations advocate for a more nuanced classification, that distinguishes between the early follicular, late follicular, ovulatory, and mid-luteal phases (Figure 1) (Elliott-Sale et al., 2021). 2) How can these phases be identified in a valid and reliable way? Several systematic reviews in recent years have highlighted a lack of methodological consistency in determining menstrual cycle phases across studies, noting considerable variability in the validity of commonly used approaches (McNulty et al., 2020; Meignié et al., 2021). Recently, Elliot-Sale et al. expressed their concern about an emerging trend toward assuming or estimating cycle phases rather than testing ovarian hormones to characterize the phases (Elliott-Sale et al., 2025). Methods such as calendar-based counting, basal body temperature tracking, and urinary luteinizing hormone (LH) testing are frequently used, yet each method has specific limitations in terms of precision and reliability, as discussed in detail earlier (Schlie et al., 2025). The most widely accepted and scientifically recommended approach is to measure serum concentrations of 17ß-estradiol (E2) and progesterone (P4), ideally in combination with the detection of the LH surge, in order to accurately confirm ovulation and determine the specific phases of the menstrual cycle (Elliott-Sale et al., 2021). Importantly, serum hormone analysis also enables the identification of anovulatory cycles and luteal phase deficiency–a critical consideration in exercise endocrinology. A luteal phase is considered physiologically adequate only if serum P4 reaches a threshold of at least 5 ng/mL (or 16 nmol/L) during the mid-luteal phase. If this threshold is not met, the cycle may be classified as luteal-deficient, even if menstruation occurs. This is particularly relevant in sport science, where the unwitting inclusion of women with such atypical cycles can lead to significant distortions in the analysis. Depending on the research question and study design, it may be necessary to exclude anovulatory or luteal-deficient cycles to ensure valid interpretation of hormone-performance relationships. While the above-mentioned P4 threshold is well-established to distinguish luteal-competent and -deficient cycles, no standardized cut-off values exist for E2 in this context. Instead, researchers typically refer to phase-specific reference ranges to interpret E2 concentrations and examine relative changes between menstrual phases. As discussed by Elliott-Sale et al., E2 values should be used descriptively, not as diagnostic thresholds (Elliott-Sale et al., 2021).

Although it is highly recommended, the serum hormone analysis has practical limitations, particularly in applied or field-based settings. High-frequency venous sampling within, sometimes, narrow timeframes requires strict compliance from the participants and the presence of qualified medical personnel to collect blood samples. These factors significantly limit the feasibility of this approach in non-clinical environments such as sports teams or during military training operations. In contrast, capillary blood sampling–which is commonly used for lactate testing in sport diagnostics–could provide a flexible, low-cost, and field-compatible alternative. If proven effective, capillary sampling would enable sport scientists and other non-medical staff to independently collect hormonal data, store the samples frozen, and later analyze them (or have them analyzed) using standard assays such as the enzyme-linked immunosorbent assay (ELISA).

Previous studies have explored the use of dried blood spot (DBS) or small-volume capillary blood drop testing for hormonal assessments. In DBS sampling, volumes of approximately 5–20 µL wet blood are transferred onto absorbent paper and left to dry at room temperature (Evans et al., 2015). As these methods typically rely on minimal sample volumes–namely, single drops of capillary blood–they are widely considered to have limited analytical scope and sensitivity (Li and Tse, 2010). Some studies however describe DBS sampling as a viable option for monitoring hormone fluctuations such as those occurring throughout the menstrual cycle, particularly when analyzed via liquid chromatography-tandem mass spectrometry (LC-MS/MS), a method known for its high sensitivity and specificity (Salamin et al., 2021). LC-MS/MS is however technically demanding and costly, requiring highly specialized laboratory infrastructure and expertise (Biotrial, 2025). This makes it less feasible for routine use in applied settings such as sport and exercise science, where budget constraints, limited access to clinical laboratories, and the need for rapid implementation are common. Edelman et al. performed an ELISA-based hormone analysis using DBS samples. They reported very high correlations between follicle-stimulating hormone, LH, and P4 concentrations in DBS samples compared to venous samples. However, they concluded that DBS sampling was less accurate for E2 (Edelman et al., 2007). Their findings suggest that low physiological levels of E2 may fall below the detection threshold or are affected by matrix-related interference when using very small sample volumes. To date, to the best of our knowledge, there is a lack of research comparing E2 and P4 concentrations in larger-volume capillary blood samples, such as those collected via lancet-assisted sampling of approximately 200 µL of blood from the earlobe, with samples obtained from venipuncture. Using such larger-volume capillary samples may offer distinct advantages as it allows for more robust standard laboratory analysis (e.g., ELISA), while maintaining the flexibility and simplicity of field-based collection.

The aim of the present study was to develop a practical and scalable approach to identify the individual cycle phases in women in tactical professions. We evaluated a capillary sampling technique that uses blood samples from the earlobe large enough to obtain serum for ELISA analysis. This hormone analysis may be performed by trained sport scientists or processed in standard diagnostic laboratories. The study aimed to answer the research question: How accurate is the determination of E2 and P4 concentrations in blood serum using larger-volume capillary blood sampling for ELISA analysis when compared to venipuncture? If validated, this approach could eliminate the need for venipuncture and medical personnel while still enabling hormonally accurate menstrual cycle tracking in field-based research.

## 2 Materials and methods

**FIGURE 2:**
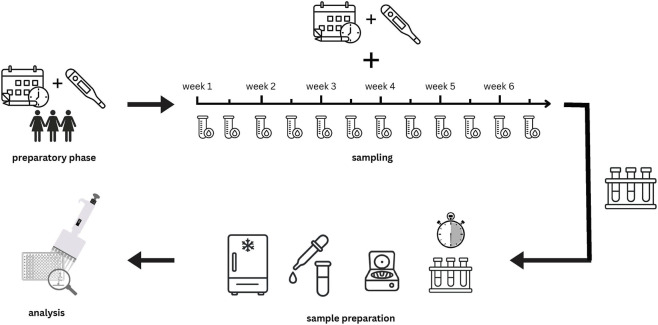
Graphical study protocol: 1. Preparatory phase: cycle tracking with menstrual calendars and LH test strips: 2. Sampling phase: over 6 weeks, blood samples were taken twice a week in pairs (capillary and venous); 3. Sample preparation: samples were left to clot for 30 min and then immediately centrifuged. Serum was then pipetted, and all samples were frozen at-80 °C; 4. Analysis: ELISA.

This study followed a longitudinal design and was conducted at the University of the Bundeswehr Munich. Twice weekly over the duration of one individual menstrual cycle, two blood samples were obtained from 12 naturally menstruating female soldiers: 1) a venous blood sample from the antecubital vein and 2) a capillary blood sample from the earlobe. Subsequently, the concentrations of E2 and P4 from both types of blood samples were analyzed by means of an ELISA. Before the start of the blood measurements, all participants tracked their menstrual cycle for at least 4 weeks by using LH-measurement strips and filling in a menstrual calendar. A study protocol is displayed in Figure 2.

This study was conducted in accordance with the Declaration of Helsinki and approved by the Ethics Committee of the University of the Bundeswehr Munich, Germany (EK UniBw M 25–10). All participants provided informed consent before study participation.

### 2.1 Subjects

Recruitment was conducted at the University of the Bundeswehr Munich from February until March 2025 by word-of-mouth. Participants fulfilled the inclusion criteria if they were a) female b) between the age of 18–35, c) occupational soldiers, d) eumenorrheic, classified as a minimum of nine cycles per annum and a cycle duration of 21–35 days, e) free from chronic or acute cardiovascular health issues, and f) willing to volunteer for screening visits under a pseudonym for the duration of one menstrual cycle. Females were not eligible if they were using any form of hormonal contraception 3 months prior to recruitment. 15 participants were initially recruited for baseline screening.

### 2.2 Menstrual cycle tracking and blood sampling

Before the start of the blood sampling, all participants tracked their menstrual cycle for at least 4 weeks by means of a menstrual calendar, in which they filled in the onset and the duration of menstrual bleeding. By this, the cycle length and the approximate timing of ovulation could be calculated for each individual. In addition, all participants used urinary LH-measurement strips from day eight of the cycle until a positive test occurred, to confirm ovulation. After this preparatory phase, venous and capillary blood samples were taken from all participants twice a week until they had completed one full menstrual cycle. This ultimately involved taking blood samples over a period of 6 weeks. In addition, the participants continued the menstrual calendar and LH-measurement. The weekly blood sampling was originally planned for Mondays and Thursdays. Due to public holidays, military commitments, and illness, individual adjustments had to be made in some cases (e.g., blood sampling on Tuesday and Thursday).

During the blood collection days, participants arrived at the medical center of the university in a fed state without having done any strenuous exercise in the morning. All blood samples were taken between 9 and 11 a.m. First, a venous blood sample of approximately 4 mL volume was drawn from the antecubital vein by the military doctor into a whole blood collection tube with coagulation factor (S-Monovette® Serum Gel CAT, 4 mL, Sarstedt AG and Co. KG, Nümbrecht, Germany). Subsequently, the earlobe of the participants was punctured with a 1.8 mm deep safety lancet (Sarstedt AG and Co. KG, Nümbrecht, Germany) by a trained sports scientist and a capillary blood sample of 100–250 µL volume was drawn into microvettes with coagulation factor (Sarstedt AG and Co. KG, Nümbrecht, Germany). The puncture site was wiped, and the first drop of blood was discarded to prevent hemolysis and contamination with interstitial fluid, after which the subsequent drops were collected into serum microtubes. Care was taken to avoid applying too much mechanical pressure on the earlobe, in order to prevent hemolysis and contamination of the capillary samples.

### 2.3 Sample preparation and ELISA

After blood collection, samples were allowed to clot for 30 min at room temperature and then centrifuged at 2500 × g for 10 min at 20°, identical to the procedure used for venous blood. The same coagulation and centrifugation protocols were applied to minimize potential matrix effects as reported by Rowland et al. (2025). Therefore, both matrices represent true serum. Serum was separated and stored at – 80 °C until analysis. To quantify progesterone and estradiol concentrations, both venous and capillary serum samples were analyzed using the Human Progesterone ELISA Kit (Abcam, Danaher Corporation, Washington DC, United States) and the Human Estradiol E2 ELISA Kit (Invitrogen, Life Technologies GmbH, Darmstadt, Germany), respectively, following the manufacturer’s instructions. For each measurement, 10 µL of serum from venous or capillary sample was diluted with 40 µL of phosphate-buffered saline, resulting in a 1:5 dilution ratio. Diluted samples were then transferred into pre-coated microplate wells in a 96-well plate in duplicate. The ELISA plates were placed in a Magellan ELISA Reader (Tecan Group Ltd., Switzerland) and incubated for 1 h at 37 °C, ensuring stable assay conditions across all samples. After the incubation period and subsequent washing steps, optical density was measured at the recommended wavelength of 450 nm. Hormone concentrations were calculated by interpolation from the standard curve generated for each plate. All paired samples (venous and capillary) were analyzed within the same assay run to minimize inter-assay variability. According to the manufacturer’s specifications, the assay’s detection limit was 0.05 ng/mL for progesterone and 10.6 pg/mL for estrogen. The assay range was 0.2–40 ng/mL for progesterone and 25–2500 pg/mL for estradiol. The intra-assay coefficients of variation (CV) for P4 and E2 were ≤4% and 11%, respectively. The inter-assay CV for P4 and E2 were ≤9.3% and 8.9%, indicating acceptable analytical sensitivity and precision for hormonal quantification.

### 2.4 Statistical analysis

To assess the agreement between capillary and venous hormone concentrations across the menstrual cycle, three complementary statistical approaches were applied. First, a repeated measures Bland–Altman analysis with mixed effects was conducted to examine the mean difference (bias) and limits of agreement (LoA) between both measurement methods while accounting for within-subject variability across the repeated sampling time points (Parker et al., 2020). Differences between capillary and venous values were plotted against their mean for each paired observation. Second, the Concordance Correlation Coefficient (CCC) according to Lin (1989) was calculated to quantify overall agreement, combining both precision (Pearson correlation) and accuracy (closeness to the 45-degree line of perfect concordance) (Lin, 1989). The CCC was reported with its 95% confidence interval and supplemented by the bias correction factor (C_ß_), which reflects the degree of systematic deviation between the two methods. Lastly, to account for the non-independence of repeated measurements within participants, a repeated-measures correlation (rmcorr) analysis was conducted to quantify the within-subject association between capillary and venous hormone concentrations (Bakdash and Marusich, 2017). Statistical analyses were performed using R (version 2024.12.1 + 563) with the nlme package (version 3.1–147) (Pinheiro et al., 2025) and the epiR package (Ste et al., 2025).

## 3 Results

Three of the 15 initially recruited participants were excluded as they missed out on more than six sampling appointments across the 6 weeks sampling timeframe. The remaining 12 subjects volunteered in at least six blood sampling appointments during one individual menstrual cycle and were able to provide complete menstrual calendars and LH-measurements across the study duration. No subject showed menstrual cycle irregularities in the form of anovulatory or luteal phase deficient cycles. All reported a positive LH peak and a mid-luteal P4 concentration of at least 5 ng/mL.

### 3.1 Descriptive statistics

The baseline characteristics of all participants are listed in Table 1. Their age ranged from 21 to 31 years. On average, the participants had a menstrual cycle of 28.3 days, with ovulation occurring on day 16.6 (Table 1). All participants were asked to provide blood samples at 12 appointments during the 6-week study period. Once an individual cycle was complete, participants were no longer required to attend further tests. On average, the women attended 8.3 appointments for blood sampling.

**TABLE 1:** Anthropometry and demographics of the n = 12 female participants.

	All participants (n = 12)	Minimum, maximum
Age (y)	24.4 ± 2.9	21, 31
Height (cm)	171.2 ± 7.8	155, 180
Weight (kg)	71.7 ± 9.6	58, 92
BMI (kg/m^2^)	24.4 ± 2.2	20.5, 28.7
Menstrual cycle length (days)	28.3 ± 3.6	22, 35
Timepoint of positive LH test/ovulation (day of MC)	16.6 ± 4.7	8, 24
Measurement timepoints within one MC	8.3 ± 1.8	6, 12
Progesterone concentration venous samples (ng/mL)	6.9 ± 8.6	0.6, 38.1
Progesterone concentration capillary samples (ng/mL)	5.3 ± 6.2	0.3, 25.4
17ß-estradiol concentration venous samples (pg/mL)	100.7 ± 64.7	3.8, 325.9
17ß-estradiol concentration capillary samples (pg/mL)	101.1 ± 62.4	17.4, 308.6

Values expressed as mean ± SD; BMI: body mass index, MC: menstrual cycle.

Progesterone concentration in the venous samples was on average 1.6 ng/mL higher compared to the capillary samples. Estradiol concentrations in the venous samples were on average 0.4 pg/mL lower compared to the capillary samples (Table 1). Table 2 displays the differences in venous and capillary E2 and P4 concentrations for the respective phases of the menstrual cycle–the early follicular (EF), late follicular (LF) and mid-luteal (ML) phase. However, it should be noted that the study design, with blood samples taken every 2–3 days, is only of limited suitability for determining the actual phases. Nevertheless, recent methodological recommendations for cycle phase determination were used as a basis here (Elliott-Sale et al., 2021).

**TABLE 2:** Venous and capillary concentrations of P4 and E2 during the estimated menstrual cycle phases.

	All participants (n = 12)	Minimum, maximum
Early follicular phase		
P4 venous samples (ng/mL)	1.75 ± 0.76	0.61, 3.37
P4 capillary samples (ng/mL)	1.59 ± 0.76	0.31, 2.82
E2 venous samples (pg/mL)	49.67 ± 13.45	31.21, 78.43
E2 capillary samples (pg/mL)	42.73 ± 12.26	25.79, 68.01
Late follicular phase		
P4 venous samples (ng/mL)	1.91 ± 1.10	0.61, 4.39
P4 capillary samples (ng/mL)	1.53 ± 0.84	0.50, 3.23
E2 venous samples (pg/mL)	217.52 ± 74.78	100.71, 325.87
E2 capillary samples (pg/mL)	213.49 ± 59.39	120.97, 308.56
Mid luteal phase		
P4 venous samples (ng/mL)	20.24 ± 9.55	5.30, 38.12
P4 capillary samples (ng/mL)	14.70 ± 6.79	5.00, 25.41
E2 venous samples (pg/mL)	122.31 ± 38.49	73.63, 192.73
E2 capillary samples (pg/mL)	143.78 ± 57.83	73.11, 301.00

### 3.2 Agreement of progesterone concentrations in capillary and venous blood samples

**FIGURE 3:**
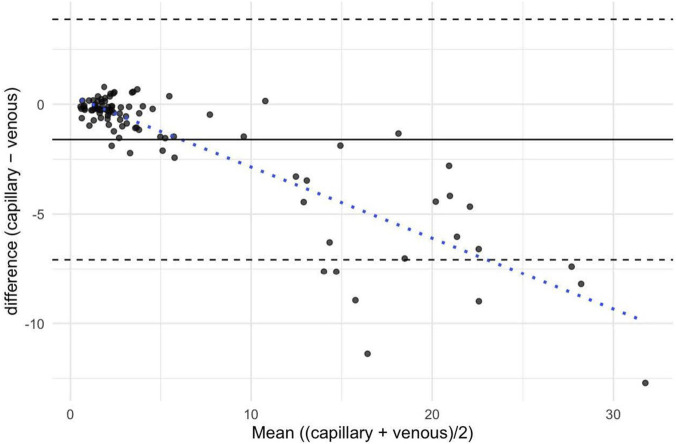
Repeated-measures Bland-Altman plot with Mixed Effects for progesterone. Differences between capillary and venous progesterone concentrations were plotted against their mean for each paired observation. The solid black line indicates the mean bias (−1.60), while the dashed lines represent the 95% limits of agreement (−7.08, +3.87). The blue dotted line depicts the proportional bias across the range of mean concentrations.

Agreement between capillary and venous P4 concentration was evaluated using a repeated-measures Bland-Altman with mixed effects and the CCC. For progesterone, the mean bias was −1.60 ng/mL, suggesting that capillary values tended to be slightly lower than venous values. The 95% LoA ranged from −7.08 to +3.87, with a total standard deviation of 2.79. The Bland–Altman plot revealed a trend towards increasing negative bias at higher mean P4 concentrations, suggesting the presence of proportional bias. Overall, agreement between capillary and venous measurements was acceptable at lower hormone levels but decreased with increasing concentrations (Figure 3).

The calculated CCC was 0.911 (95% confidence interval (CI): 0.887–0.930), indicating good agreement between the two measurement methods. The bias correction factor (C_ß_) was 0.929, suggesting a low level of systematic deviation (Table 3).

**TABLE 3:** Statistical analysis–Mean bias and Concordance Correlation Coefficient.

Progesterone (P4)	
Mean bias	−1.60 ng/mL
Concordance correlation coefficient	0.911 (CI: 0.887 to 0.930)
Bias correction factor (C_ß_)	0.929
17ß-Eststradiol (E2)	
Mean bias	−0.34 pg/mL
Concordance correlation coefficient	0.919 (CI: 0.882 to 0.945)
Bias correction factor	0.999

*CI: 95 % confidence interval of the concordance correlation coefficient.

The repeated-measures correlation demonstrated a strong within-subject association between capillary and venous progesterone values (rmcorr = 0.981), further supporting the consistency of both sampling methods across repeated measurements (Figure 5A).

### 3.3 Agreement of estradiol concentrations in capillary and venous blood samples

**FIGURE 4:**
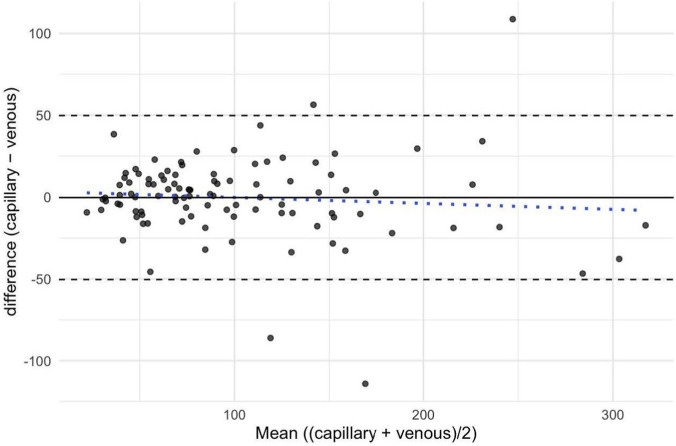
Repeated-measures Bland-Altman plot with Mixed Effects for estradiol (E2). Each point represents one paired capillary-venous measurement across subjects and time points. The solid black line indicates the mean bias (−0.34), while the dashed lines denote the 95% limits of agreement (50.47, 49.80). The blue dotted line represents the proportional bias.

For the capillary and venous E2 concentration, the mean bias was −0.34 pg/mL, indicating minimal systematic difference between capillary and venous concentrations. The 95% LoA ranged from −50.47 to +49.80, corresponding to a total standard deviation of 25.58. This wide range of agreement suggests that while the overall bias was minimal, the variability between the two sampling methods increased considerably at higher concentrations. Visual inspection of the Bland–Altman plot confirmed that most differences were centered around zero, with a few outliers at higher mean values, but no clear proportional bias was evident (Figure 4).

The calculated CCC was 0.919 (95% CI: 0.882–0.945), indicating very good agreement between the two measurement methods. The bias correction factor was 0.999, suggesting a negligible level of systematic deviation (Table 3).

**FIGURE 5:**
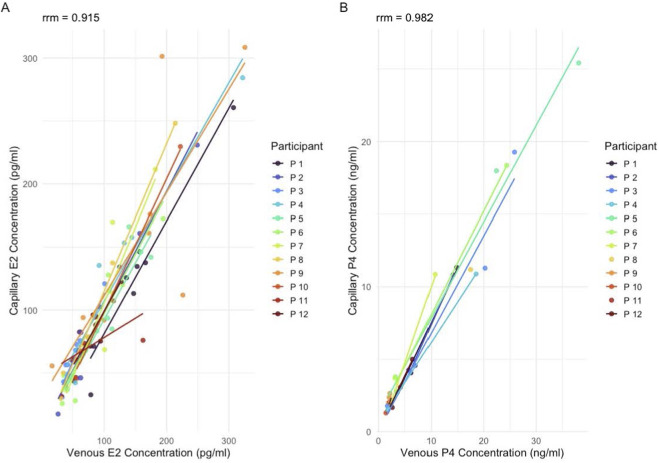
Repeated-measures correlation (mcorr) between venous and capillary hormone concentrations. **(A)** Estradiol (E2) concentrations (pg/mL) and **(B)** progesterone (P4) concentrations (ng/mL). Each color represents an individual participant (n = 12), with subject-specific regression lines illustrating within-participant associations across repeated measurements.

The repeated-measures correlation demonstrated a strong within-subject association between capillary and venous E2 values (rmcorr = 0.915), further supporting the consistency of both sampling methods across repeated measurements (Figure 5B).

## 4 Discussion

This study investigated the validity of measuring two major female sex hormones, P4 and E2, in larger-volume capillary blood samples obtained from the earlobe, compared to conventional venous blood samples drawn from the antecubital vein. The primary aim was to evaluate whether capillary-derived hormone concentrations provide a reliable basis for menstrual cycle phase classification, particularly in comparison to the current gold standard of venous sampling. The findings demonstrate that capillary sampling offers a practical and physiologically sound alternative to venous blood collection for monitoring both P4 and E2. This is especially relevant in field-based or operational environments, such as elite sports or military settings, where access to clinical infrastructure is limited and the need for frequent, minimally invasive sampling is high.

### 4.1 Agreement of progesterone and estradiol measurements

The comparison of P4 concentrations revealed a systematic bias, with capillary values being on average 1.60 ng/mL lower than venous values. This difference was consistent across participants and confirmed by the Bland–Altman analysis, which indicated increasing negative deviation at higher P4 concentrations, suggesting a proportional bias. Importantly, the agreement between the two methods was acceptable at lower hormone levels, with the majority of paired observations falling within the 95% LoA. From a practical perspective, this systematic underestimation may be considered favorable, particularly when applying the established ≥5 ng/mL mid-luteal threshold to define luteal sufficiency (Elliott-Sale et al., 2021). A capillary P4 measurement meeting or exceeding this cut-off is likely to correspond to an even higher venous value, thereby minimizing the risk of false-negative classification. This is especially relevant for studies aiming to distinguish ovulatory from anovulatory or luteal-deficient cycles. The calculated CCC of 0.911 with a bias correction factor of 0.929 further supports the high level of agreement between capillary and venous sampling. While to date no published study has directly compared venous with larger-volume capillary samples for sex hormone analysis, our findings align with previous work using DBS methods. For example, Edelman et al. reported a correlation coefficient of r = 0.82 between DBS-derived and venous P4 values, supporting the use of minimally invasive sampling for reproductive hormone monitoring (Edelman et al., 2007). Although the observed proportional bias suggests that capillary P4 concentrations may underestimate actual venous values, this limitation is unlikely to compromise cycle phase classification in most applied settings. Once the ≥5 ng/mL threshold is exceeded, the exact P4 concentration typically carries limited additional diagnostic or practical relevance. Thus, the method appears well-suited for menstrual cycle tracking in field-based research and performance contexts.

The comparison of E2 concentrations showed very high agreement between capillary and venous samples, with a mean bias of −0.34 pg/mL and a CCC of 0.919. This minimal difference indicates that capillary sampling provides an accurate and reliable estimate of venous estradiol levels. While the 95% LoA were relatively wide (−50.47 to +49.80 pg/mL), this range must be interpreted in the context of the measurement unit: estradiol is quantified in picograms per milliliter (pg/mL), which is 1,000 times smaller than the nanogram unit used for progesterone. Hence, even large numerical deviations correspond to physiologically small differences. Visual inspection of the Bland–Altman plot confirmed that most paired observations were clustered around zero, with only a few outliers at higher mean concentrations. No systematic or proportional bias was evident. Unlike P4, E2 has a broad physiological fluctuation across the menstrual cycle and does not rely on a fixed and universally accepted diagnostic threshold for menstrual phase classification. Instead, its interpretation depends on relative phase-dependent changes, such as the pre-ovulatory surge and late-luteal decline of E2 (Elliott-Sale et al., 2021). Consequently, in most applied settings in sport and exercise science, small absolute differences between the two sampling methods are of limited practical relevance, provided that the temporal fluctuations of E2 are accurately captured. The present findings indicate that capillary sampling can accurately reflect these physiological fluctuations, making it suitable for longitudinal or high-frequency monitoring of E2 in applied research settings. These results are consistent with previous studies using DBS sampling, which also reported a strong correlation between DBS and venous E2 concentrations (*r* = 0.70), albeit slightly less strong than for P4 (Edelman et al., 2007).

While no previous study has compared larger-volume capillary samples with venous serum for hormonal analysis, recent findings by Rowland et al. highlight the importance of the sample matrix (that is, differences in the composition of plasma, serum, or capillary blood) that can affect hormone measurements. In their comparison of venous plasma and serum, mean concentrations of E2 and P4 were 44.2% and 78.9% higher in plasma, respectively (Rowland et al., 2025). These substantial differences underline that even within venous sampling, the choice of matrix can systematically influence hormone values, and must be considered when interpreting thresholds or comparing across studies.

Compared to DBS sampling, which relies on minimal sample volumes (typically single drops of blood), larger-volume capillary sampling (100–250 µL) allows for more robust hormone quantification using standardized ELISA, without the need for more complex and costly methods such as LC-MS/MS. Salivary sampling is another method that would supposedly offer clear advantages over capillary measurement. Here, large samples can be collected with a non-invasive method, which is advantageous from a logistical point of view. Saliva analysis is however limited by the substantially lower concentrations of steroid hormones in saliva compared to blood that may reduce measurement reliability. Thus, capillary sampling strikes a balance between analytical accuracy and field compatibility for endocrine monitoring in female athletes.

### 4.2 Methodological considerations and limitations

Several methodological aspects and limitations of this study should be considered when interpreting the results. One frequently mentioned challenge in capillary blood sampling is the increased risk of sample contamination, particularly due to hemolysis, tissue fluid admixture, or improper technique during collection (Royal et al., 2022). This risk is especially relevant when excessive pressure is applied to the tissue of the puncture site. In the present study, we addressed this issue by employing trained personnel and using standardized procedures. For instance, the first drop of blood from the draw was always wiped away before the actual sample was taken to minimize contamination. We used lancets with a shallow penetration depth of 1.8 mm to collect 100–250 µL of blood from the earlobe. It is reasonable to assume that using lancets with slightly greater penetration depth (e.g., ≥2.0 mm) could further reduce the need to apply pressure on the tissue. Thereby, the likelihood of hemolysis and tissue trauma would be reduced–particularly in settings where researchers have limited experience with capillary sampling. Another practical limitation relates to the processing of capillary samples. While the serum derived from capillary blood samples can be frozen and stored for later batch analysis via ELISA, there are initial handling steps–namely, centrifugation and serum pipetting–that must be performed promptly after sample collection. This requires a minimum level of laboratory infrastructure and technical skill at the sampling site. Compared to DBS methods, which allow for passive drying and room temperature storage, capillary serum sampling is therefore more time-sensitive and logistically demanding. Nevertheless, the feasibility is significantly higher compared to repeated venous sampling in field-based environments. Another limitation is the supposedly low sample size of this study (n = 12). However, the number of paired data points per subject (≥6 measurement timepoints per cycle) provided sufficient statistical power to evaluate the agreement across a broad hormonal range. All participants exhibited a clear LH surge and luteal P4 concentrations exceeding the 5 ng/mL threshold, indicating ovulatory and luteal-competent cycles throughout the study. This is particularly interesting, since the twice-weekly sampling schedule applied in this study likely failed to capture the actual peak hormone concentrations (e.g., pre-ovulatory E2 surge or mid-luteal P4 maximum), preventing a precise assessment of capillary-venous agreement at these physiologically critical timepoints. Given the relatively high prevalence of anovulatory or luteal-phase deficiency reported in young, athletic, eumenorrheic women (Recacha-Ponce et al., 2025; Manore, 2002) this homogeneity may reflect a selection bias, timing effects, or simply the limited sample size, and should be interpreted with caution. Another limitation concerns the systematic underestimation of P4 in the capillary compared to the venous samples as shown in the Bland-Altman plot (Figure 3). Although the proportional bias observed is unlikely to affect classification of subjects when P4 concentrations are well above the 5 ng/mL threshold, uncertainty may arise near this cut-off, where a capillary value of 4–6 ng/mL could correspond to venous concentrations both below and above the criterion for luteal sufficiency. Lastly, the timing of blood collection was not pre-aligned to specific cycle phases. This design was chosen intentionally, as this study represents a feasibility trial that aimed to assess the logistical feasibility of high-frequency blood sampling for cycle tracking under real-world conditions in a tactical population.

Future research should address several key aspects to strengthen the validation of capillary hormone sampling. First, more precise phase-targeted or adaptive sampling protocols are needed to capture hormonally critical timepoints, ideally in larger sample sizes. Second, incorporating longer preparatory tracking phases (e.g., 2-3 cycles of LH testing and calendar-based monitoring) could improve temporal alignment between sampling and target cycle phases. Lastly, studies should include women with atypical cycles (such as anovulatory and luteal-phase deficient cycles) and assess the method’s sensitivity and specificity, specifically near the clinical threshold for luteal-sufficiency.

In terms of feasibility, adherence in the present study was high: 12 of the 15 initially enrolled participants completed at least six capillary and venous sampling visits within a single menstrual cycle. This pattern suggests that repeated capillary blood collection is logistically feasible and can be implemented in non-clinical settings without major organizational barriers. However, we acknowledge that our definition of feasibility - operationalized primarily through adherence - represents a relatively narrow perspective. Importantly, we did not collect participant-reported outcomes that would allow a more comprehensive assessment of acceptability, such as perceived burden, discomfort, time demands, or overall willingness to engage in repeated sampling. The absence of such data is a significant limitation, as feasibility critically depends not only on the ability of participants to complete procedures but also on how tolerable and acceptable they perceive those procedures to be. Future studies should therefore incorporate validated instruments to assess participant burden, such as the *Perceived Research Burden Assessment* (Lingler et al., 2014), to capture subjective experiences systematically. Including these measures would enable a more holistic evaluation of feasibility and help refine repeated capillary blood sampling protocols in ways that balance scientific rigor with participant wellbeing in real-world research contexts.

### 4.3 Conclusion and future perspectives

This study is the first to assess the validity of larger-volume capillary blood sampling for the quantification of P4 and E2 in comparison to venous sampling across a complete menstrual cycle in women from tactical professions. The findings demonstrate a high level of agreement between both sampling methods, supporting the use of capillary serum as a practical, minimally invasive, and logistically feasible alternative for hormone monitoring in field-based settings.

The ability to collect capillary blood samples without the need for medically trained personnel significantly lowers operational barriers in applied environments such as team sport, military training, or performance diagnostics. Moreover, the option to store frozen capillary serum for later analysis adds further flexibility, particularly where repeated sampling is needed but laboratory infrastructure is limited. Based on these findings, capillary sampling may help to facilitate more hormonally informed research designs and encourage the inclusion of female participants in studies where traditional venous sampling would be impractical or impossible. Future research should aim to validate this approach in larger cohorts, and across multiple menstrual cycles. Studies in elite athletic populations are warranted to evaluate the method’s robustness under high-performance conditions and to explore its potential in performance monitoring, injury prevention, and recovery management.

## Data Availability

The datasets presented in this study can be found in online repositories. The names of the repository/repositories and accession number(s) can be found below: https://osf.io/5bfnq/overview?view_only=0be35c65b3864e95bcfeb2732907abb9.
